# Identification and phylogenetic analysis of the complete mitochondrial genome of Mediterranean buffalo (Artiodactyla: Bovidae)

**DOI:** 10.1080/23802359.2020.1714503

**Published:** 2020-01-20

**Authors:** Qian Lin, Meng-Wei Li, Yan-Zhou Wang

**Affiliations:** a Institute of Bast Fiber Crops, Chinese Academy of Agricultural Sciences, Changsha, China;; b Buffalo Research Institute, Chinese Academy of Agricultural Sciences and Guangxi Zhuang Nationality Autonomous Region, Nanning, China

**Keywords:** Mediterranean buffalo, mitochondrial genome, phylogenetic analyses

## Abstract

Mediterranean buffalo (*Bubalus bubalis* Linnaeus, 1758 breed Mediterranean, MEB) is one of the best milk-producing breeds in river-type buffaloes in the world. It is the first time that the complete mitochondrial genome sequence of the MEB was reported. The total length of the mtDNA is 16,357 bp, It contains the typical structure, including 22 transfer RNA genes, 2 ribosomal RNA genes, 13 protein-coding genes and 1 non-coding control region (D-loop region). The overall composition of the mtDNA was estimated to be 33.10% for A, 26.44% for T, 26.57% for C and 13.89% for G, in the order A > C > T > G feature occurs in the MEB. Phylogenetic analyses using N-J computational algorithms showed that the analyzed 18 ruminantia species are divided into four major clades: Bovidae, Cervidae, Giraffidae and Atilocapridae. In addition, our work confirmed that MEB and Nili-Ravi buffalo (NRB) have a close genetic relationship with fellow tribal members Murrah buffalo. Meanwhile, we also found that MEB and NRB have highly similar genetic relationship.

Mediterranean buffalo (*Bubalus bubalis* Linnaeus, 1758 breed Mediterranean, MEB) is one of the best milk-producing breeds in river-type buffaloes in the world (Chen et al. 2015). In this study, we newly determined the complete mitochondrial genome of MEB, and the specimens were collected from the adult individuals of MEB at its culturing farm in Nanning city (22°90′29.27″N and 108°36′07.38″E), Guangxi Zhuang Nationality Autonomous Region, China on September 2019. And the specimens were stored at −80 °C in the National Buffalo Resources Specimen Library of China (Buffalo Research Institute, Chinese Academy of Agricultural Sciences and Guangxi Zhuang Nationality Autonomous Region, Nanning, China) with a catalog number of MEB20190901. Total genomic DNA was extracted from the whole blood specimen of a single individual using the EasyPure Kit of Genomic DNA (Transgen Biotech, Beijing, China). Whole mitochondrial genome was amplified with 11 pairs of primers and sequenced by BioSune Biotech (Shanghai, China). DNA sequence was analyzed using DNAStar.Lasergene.v7.1 software (Madison, WI), tRNA Scan-SE1.21 software (Lowe and Eddy 1997) and DOGMA software (Wyman et al. 2004).

The total length of the mtDNA is 16,357 bp, It contains the typical structure, including 22 transfer RNA genes, 2 ribosomal RNA genes, 13 protein-coding genes and 1 non-coding control region (D-loop region) (GenBank accession No. MN756622). The overall composition of the mtDNA was estimated to be 33.10% for A, 26.44% for T, 26.57% for C and 13.89% for G, in the order A > C > T > G feature occurs in the MEB. Besides the ND2, ND3 and ND5 initiation codon are ATA, ND4L is GTG, and the rest of the proteins are ATG. All these genes have 13 spaces and 10 overlaps both in the length of 1–40 bp. These genes had four types of termination codons, including TAA, TAG, AGA and an incomplete termination codon ‘‘T– –’’. ‘‘T– –’’ is the 5’ terminal of the adjacent gene (Anderson et al. 1981). Among 13 protein-coding genes, the longest one was ND5 gene (1821 bp), which was located between the tRNA^Leu^ and ND6, and the shortest one was ATPase8 gene (201 bp), which was located between the tRNA^Lys^ and ATPase6. The lengths of 12S rRNA and the 16S rRNA were 957 bp and 1569 bp. And deduced 22 tRNA genes were distributed in rRNA and protein-coding genes, ranging from 60 to 75 bp in size. The mitochondrial DNA D-loop region of the MEB was located between tRNA^Pro^ and tRNA^Phe^ with a length of 926 bp.

Phylogenetic analysis was performed using the complete mitochondrial DNA sequences of 18 ruminantia species. Each of the sequence dataset was aligned by ClustalX (Thompson et al. 1997) and analyzed by neighbor-joining (N-J) in MEGA 4.0 (Tamura et al. 2007), and bootstrap analysis was performed with 100 replications. An N-J tree showed that the analyzed species are divided into four major clades (Figure 1). Bovidae makes up the first lineage, which is sister to the second group, Cervidae; Giraffidae forms the third group and is sister to Bovidae and Cervidae. The lineage consisting of these three groups in turn is sister to the fourth clade, Atilocapridae. In addition, our work confirmed that MEB and Nili-Ravi buffalo (NRB) have a close genetic relationship with fellow tribal members Murrah buffalo (MB). Meanwhile, we also found that MEB and NRB have highly similar genetic relationship.

**Figure 1.**
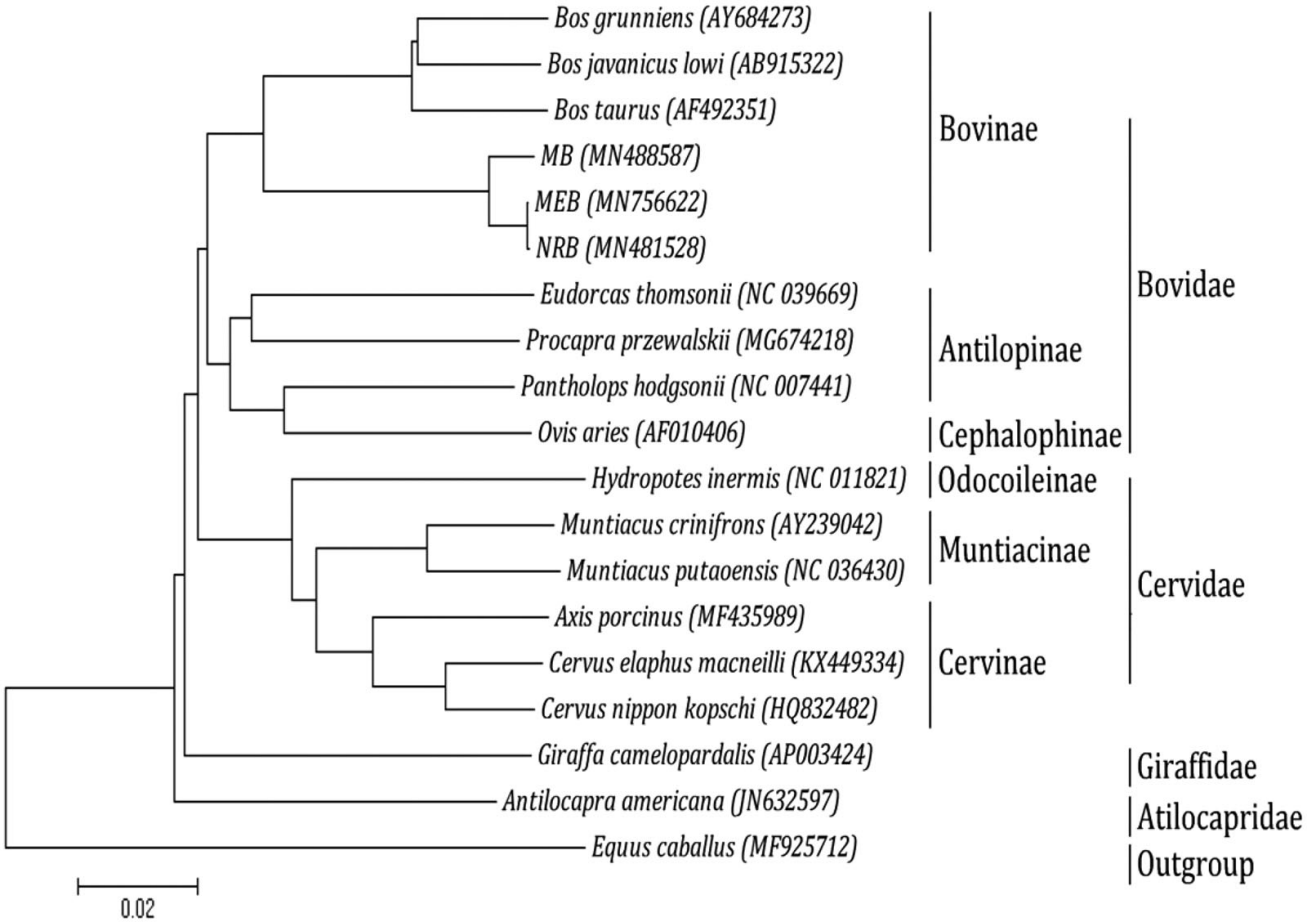
Phylogenetic analysis based on complete mitochondrial genome sequences. An N-J tree was built based on the phylogenetic analysis of 18 ruminantia species’ complete mitochondrial genomes. The mitochondrial genome sequences of the ruminantia species were obtained from the GenBank databases (Accession numbers have marked on the figure). Abbreviation of species indicates: MB: Murrah buffalo; MEB: Mediterranean buffalo; NRB: Nili-Ravi buffalo.
